# Peripheral Blood MDSCs, IL-10 and IL-12 in Children with Asthma and Their Importance in Asthma Development

**DOI:** 10.1371/journal.pone.0063775

**Published:** 2013-05-22

**Authors:** Yan-Li Zhang, Bin Luan, Xiu-Fang Wang, Jun-Ying Qiao, Li Song, Rui-Rui Lei, Wei-Xia Gao, Ying Liu

**Affiliations:** Department of Pediatrics, the Third Affiliated Hospital of Zhengzhou University, Zhengzhou, Henan, China; University of California, Riverside, United States of America

## Abstract

**Objective:**

To investigate myeloid-derived suppressor cell (MDSC) accumulation and interleukin 10 (IL-10) and interleukin 12 (IL-12) levels during the onset of asthma in both pediatric patients and mouse models, as well as their possible roles in the development of asthma.

**Methods:**

Peripheral blood samples were gathered from children with asthma attacks (attack group) and alleviated asthma (alleviated group), as well as two control groups, children with pneumonia and healthy children. The pathological characteristics of asthma in asthmatic mice, budesonide-treated asthmatic mice, and normal control mice were also evaluated by immunohistochemistry (IHC) and hematoxylin and eosin (H&E) staining.

**Results:**

MDSC accumulation and serum IL-10 levels were significantly elevated in the children with asthma compared with the budesonide-treated alleviated group, normal healthy controls, and pneumonia controls (p<0.05), whereas those in the latter three groups showed no statistical differences (p>0.05). The level of serum IL-12 in the asthmatic children was drastically reduced compared to the budesonide-treated alleviated group, healthy controls, and pneumonia controls (p<0.05), whereas the latter three groups showed no significant differences in their serum IL-12 levels. The percentage of MDSCs in children with asthma was positively correlated with the level of serum IL-10 and negatively correlated with the level of serum IL-12. The levels of MDSCs and IL-10 in asthmatic mice were significantly higher than those in the normal control mice (both p<0.05) and were reduced after budesonide treatment (both p<0.05). IL-12 expression in the asthmatic mice was significantly lower than the control and was increased upon budesonide treatment (both p<0.05).

**Conclusion:**

During the onset of asthma, the accumulation of MDSCs and the level of serum IL-10 increase, while the level of IL-12 decreases. These fluctuations may play an important role in the development of asthma.

## Introduction

Asthma is a common chronic inflammatory disease of the airways and a severe health risk for children [1]. So far, the mechanisms of asthma onset and development are still unclear, and asthma patients respond differently to current treatments. Although recent immunology studies have showed their value in understanding asthma such as immune marker-specific immunoglobulin E (IgE) and eosinophils (EOSs) in the diagnosis of asthma, so far there is lack of evidence to explain the fundamental mechanisms of asthma onset and to support targeted therapy against asthma. Currently unreliable diagnosis and excessive treatment in asthma, especially asthma in children, largely compromised the physical and mental development of children.Thus, it is important to develop novel diagnosis markers and treatment targets for asthma.

Asthma is caused by T helper type 2 (Th2)-induced reversible airway obstruction and airway hyperresponsiveness [2]. Currently, the pathogenesis of asthma is still elusive. Although cytokines, chemokines, and myeloid progenitor inhibitory factors are thought to be involved, the underlying mechanisms are still far from known [3], [4]. Recently, it was reported that IL-10 and IL-12, important cytokines secreted by helper T lymphocytes, may play important roles in the pathogenesis of asthma [5]. In the balance between type 1 and type 2 helper T lymphocytes (Th1/Th2), IL-10 enhances the differentiation of Th0 to Th2 through inhibiting the proliferation of Th1 or directly activating Th2 [6]. IL-12, which connects the innate immunity and adaptive immunity, plays an important role in regulating anti-infection immune responses and inflammation. Studies on animal models of asthma indicate that immune cells are involved in asthma development but the mechanism remains unclear [3]. MDSCs are a heterogeneous population of immature myeloid cells characterized as CD11b^+^CD33^+^. MDSCs are significantly increased in severe infection, tumor, and acute inflammation, and they negatively regulate immune functions [7], [8]. Mouse MDSCs often express both CD11b and Gr-1, or either one of the two markers depending on different mouse strains, organs, and disease status [7]. MDSCs produce high levels of IL-10 and thereby reduce the level of IL-12 under severe inflammation [8].

To better understand the function of the key cytokines and immune cells during the asthma onset and development in detail, we examined the levels of MDSCs, IL-10, and IL-12 in children and mice. We found that the accumulation of MDSCs, which was positively correlated IL-10 levels and negatively correlated with IL-12 levels, is significantly increased during the onset of asthma in both human and mice. Therefore, we propose that MDSCs play a role in asthma pathogenesis by up-regulating IL-10 and down-regulating IL-12.

## Materials and Methods

### Ethics Statement

The Medical Ethics Association of the Third Affiliated Hospital of Zhengzhou University reviewed all the protocols and approved this study. For the patient study, written consents were received from the parents of all children involved. The animal studies were approved by the Animal Ethics Committees of Third Affiliated Hospital of Zhengzhou University. Animals were housed and treated under the approved protocols. All mouse work was consistent with the requirement of the Animal Ethics Committees of Third Affiliated Hospital of Zhengzhou University. All efforts were made to minimize animal suffering.

### Patient Study

The patients were categorized into three groups: asthma group, pneumonia group and normal healthy group. The asthma group consisted of 102 children (48 males, 54 females) aged 1 to 5 years (ave = 3 years) with asthma, who visited a doctor or were hospitalized in the Third Affiliated Hospital of Zhengzhou University from January 2011 to April 2012. The asthma group was divided into two subgroups: attack group and alleviated group (budesonide-treated group). The attack group consisted of 52 children (28 males, 24 females) aged 1 to 5 years (ave = 3 years) with acute asthma onset. The alleviated group included 50 children (26 males, 24 females) aged 1 to 5 years (ave = 3 years) in their asthma alleviated phase. All the patients were confirmed according to the published diagnostic standards [9]. The healthy control group comprised of 48 normal healthy children (26 males, 22 females) aged 2 to 5 years (ave = 3 years) randomly chosen from those who had a physical examination in the hospital in the same time period. The pneumonia group comprised of 45 children (25 males, 21 females) aged 1 to 4 years (ave = 3 years) who were diagnosed with pneumonia in the same hospital in the same time period. There were no significant differences in the age and gender composition among the groups (p>0.05).

Children with diagnosed atopy and (or) parent of children with diagnosed asthma history are considered atopic factor, which constitutes 50 cases in asthma attack group, 49 in alleviated group, and 0 in the normal or pneumonia control group. Asthma is divided into four scales according to the severity of the diseases: mild, moderate, severe, and critical. The diseases in the asthma patients for this study were categorized as mild or moderate [9].

### Animal Study

30 SPF (Specific Pathogen Free) grade 5- to 6-week-old female Balb/C mice were provided by Zhengzhou University Animal Experiment Center, kept in the Experiment Center of the Third Affiliated Hospital of Zhengzhou University with free access to food and water for one week prior to the experiment. Mouse body weights ranged from 12–20 g as determined by a digital scale before the experiment. The establishment of an asthma model with the asthma group was performed as described in protocols with modifications [10]. Briefly, each mouse was injected intraperitoneally with ovalbumin (OVA)/aluminium hydroxide 0.2 mL on day 1, 8, and 15, and every other day starting from day 22 for a total of 7 doses. Mice immediately inhaled aerosol 2% OVA for stimulation for 30 min. Asthma was established in the alleviated group using the same procedure except with an additional 30 min inhalation of 1 mg (2 ml) budesonide before the stimulation. Negative control mice were treated under the same protocol as asthmatic mice except a saline solution replaced OVA. No mice from any of the three groups died during the experiments.

### Criteria for Mouse Airway Hyperreactivity

Mice displaying shortened breath, restlessness, blue face, Salivation, and incontinence upon allergen inhalation were considered having airway hyperreactivity. More severe reaction includes hypopnea or respiratory arrhythmia, paralysis, and lack of responses.

### Reagents

Human IL-10 ELISA kit (R&D, USA), Human IL-12 ELISA kit (R&D Systems Inc, Minnesota, USA), Human IL-13 ELISA kit (R&D Systems Inc, Minnesota, USA), Human IL-17 ELISA kit (R&D Systems Inc, Minnesota, USA), Human IL-4 ELISA kit (R&D Systems Inc, Minnesota, USA), Mouse NO ELISA kits (Jiang Lai Bio-Technology, Shanghai, China), anti-CD4^+^ antibody Human T-IgE ELISA Kits (R&D Systems Inc, Minnesota, USA), PE-conjugated anti-CD33 antibody (human) and APC-conjugated anti-CD11b antibody (human, both from Becton Dickinson, Heidelberg, Germany), Arginase I antibody (Santa Cruz, California, USA), APC-labeled mouse anti-human CD25 monoclonal antibody (R&D Systems Inc, Minnesota, USA), DCFH-DA (Sigma-Aldrich, Missouri, USA) purified anti-MDSCs antibody (eBioscience, San Diego, California, USA), rabbit anti-mouse IL-10 polyclonal antibody and Rabbit anti-mouse IL-12 polyclonal antibody (both from Beijing ZhongShan JinQiao Biotechnology, China), DAB and SP immunohistochemistry (IHC) staining kit (Beijing ZhongShan JinQiao Biotechnology, Beijing, China), OVA (Grade V) (Sigma-Aldrich, Missouri, USA), budesonide (AstraZeneca, Shanghai, China), 0.9% NaCl injection (Hengrui medical Co, Jiangsu, China), emulsified aluminum hydroxide (Beijing Chemical, Beijing, China), 10% neutral formalin (Shanghai Chemical medicine, Shanghai, China). Respiratory virus detection kit (Beijing People’s Hospital 262, Beijing, China).

### ELISA

ELISA was performed to detect IL-10, IL-4, IL-13, IL-17, NO and IL-12 according to the manufacturer’s instructions (R&D Systems Inc, Minnesota, USA).

### Flow Cytometric Analysis

The peripheral blood mononuclear cells (PBMCs) were incubated with PE-conjugated anti-CD33 antibody and APC-conjugated anti-CD11b antibody anti-CD4^+^ antibody Human T-IgE ELISA Kits (R&D Systems Inc, Minnesota, USA), and then detected by flow cytometry (FACS Calibur, Becton Dickinson). The data was analyzed with CellQuest software (BD Biosciences, California, USA). The nucleated cell population (R1) was gated to remove dead cells and cell debris. The two-dimensional scatter diagram displays cells in R1, with the upright fraction showing CD11b^+^/CD33^+^ cells.

### ROS Detection

The accumulation of ROS was detected with 2,7-dichlorodihydrofluorescein diacetate (DCFH-DA). Specifically, DCFH-DA was added to isolated Bone marrow mononuclear cells (BMMNCs) resuspended in PBS to a final concentration of 10 pM, incubated in 37°C for 30 mins, and then washed with PBS twice before analyzed by flow cytometry.

### H&E and IHC Staining

The procedures were performed according to the kit manufacturer instructions. The MDSCs in mouse lung tissues were stained with anti-Gr-1 antibody and scored under the microscope for yellow/brown staining. Images of Gr-1 stained IL-10, IL-12, IL-6, TNF-α, Arginase 1, and MDSCs were taken at 200× magnification and were analyzed with Image Pro Plus 6.0, where five positive areas were selected per slice and the optical density values were measured. The expression levels were represented by the mean density of 5 areas per slide.

### PAS Staining and Quantitation

Paraffin-embedded slides were deparaffinized and incubated with 1% Sodium periodate for 10 mins, and then washed with ddH_2_O for 2 min twice. The slides were then stained in Schiff staining buffer for 20 mins followed by ddH_2_O wash for 10 min, and hematoxylin nucleus staining for 10 secs followed by 20 secs of 1% hydrochloric-alcohol solution treatment. The slides were washed with running water and dehydrated with ethanol before mounted with Neutral Balsam mounting medium. To quatify PAS-positive cells, the optical density of five representative positive areas were measured. The expression levels were represented by the mean density of 5 areas per slide.

### BALF Cell Isolation

Mice were sacrificed by cervical dislocation 24 h after the last stimulation, chest opened for the following procedures. Tracheal intubation was performed with modified 22 G catheter following ligation of distal trachea and left main bronchus, and 0.3 mL PBS lavage for 3 times. BALF was collected with resorption rates over 80%. Supernatant was collected after 5 mins of centrifugation at 4°C 1500 r/min, and stored at −20°C for CD4+ Tregs detection. The cell pellets were resuspended in PBS, and the numbers of macrophages, eosinophils and neutrophils were counted. Left lung tissue was fixed with 4% Paraformaldehyde, dehydrated with ethanol, embedded with paraffin and stained with H&E staining.

### BALF Differential and Cell Count

Cell pellets were resuspended in 10 mL PBS. 10 µL solution was mixed with 10 µL white blood cell dilution buffer, followed by total cell counting with hemocytometer. Subtypes of white blood cells were scored with 50 µL Wright’s stained cell suspension under optical microscope. Each sample score 4 white blood cells, and the percentage of each subtype (eosinophils, lymphocytes, macrophages, and neutrophils) was calculated.

### Mouse Serum Isolation

Blood samples was collected from sterile retro-orbital bleeding, allowed to clot at room temperature and centrifuged for 10 mins at 2000 rpm. Serum was collected from the top of the tube and aliquot for further analysis.

### Statistical Analysis

All data were analyzed with SPSS v17.0 and were presented in the format 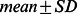. Independent sample *t* tests were done to compare the difference between groups. Pearson correlation was used to analyze the relativity with a = 0.05 as the standard for a significant difference.

## Results

### MDSCs and IL-10 are Highly Induced, while IL-12 is Significantly Reduced in Asthmatic Children

**Figure 1:**
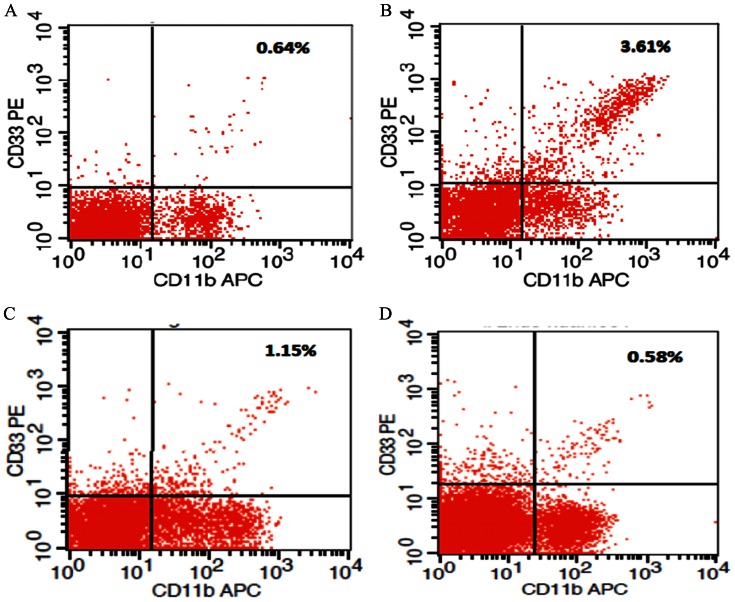
Representative flow cytometric analysis of the percentage of MDSCs in PBMCs in asthma patients, budesonide-treated asthma patients, healthy controls and pneumonia patients 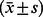. (A) Percentage of MDSCs (CD11b^+^/CD33^+^) in PBMCs in the normal group. (B) Percentage of MDSCs in PBMCs in the attack asthma group. (C) Percentage of MDSCs in PBMCs in the budesonide treated group. (D) Percentage of MDSCs in PBMCs in the pneumonia group.

**Figure 2:**
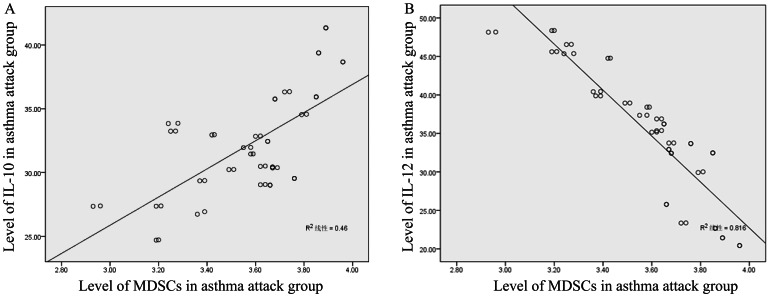
The correlation between the levels of MDSCs and the level of IL-10 and IL-12 in asthma patients. (A) The positive correlation between the level of MDSCs and the level of serum IL-10 in asthma attack patients. (B) The negative correlation between the level of MDSCs and the level of serum IL-12 in attack asthma group.

195 children that were hospitalized in the Third Affiliated Hospital of Zhengzhou University were divided into four groups: attack group, alleviated group, normal healthy group and pneumonia group. Respiratory secretion pathogen analysis showed 25 cases of Respiratory Syncytial Virus and 23 cases Rhinovirus in the asthma group, 18 cases of Respiratory Syncytial Virus and 15 cases Rhinovirus in the alleviated group, 10 cases of Respiratory Syncytial Virus and 9 cases Rhinovirus in the pneumonia group, and 0 for normal control group. We measured the accumulation of MDSCs by flow cytometry and the levels of serum IL-10 and IL-12 by ELISA. In asthma patients, the percentage of MDSCs in the peripheral blood mononuclear cells (PBMCs) and the level of serum IL-10, IgE, EOS were both significantly elevated compared to normal controls, budesonide-treated alleviated asthma patients, and pneumonia patients (p<0.05), whereas those in the latter three groups showed no statistical differences (p>0.05). The level of serum IL-12 in the asthmatic children was drastically reduced compared to the other three groups (p<0.05), whereas the other three groups showed no significant differences (p>0.05, Figure 1 and Table 1). The amount of MDSCs in peripheral blood was positively correlated the level of IL-10 in the serum of the patients with asthma attacks (r = 0.741, *P*<0.01, Figure 2A) and negatively correlated with the level of serum IL-12 (r = −0.879, *P*<0.01, Figure 2B). However, there was no correlation between MDSCs and the level of IL-10 in the alleviated group, normal control group, or the pneumonia group. Therefore, the clinical results indicate that the MDSCs immune cell and IL-10 and IL-12 cytokines are significantly regulated in asthmatic children and might play important roles during the onset and development of asthma.

**Table 1:** The percentage of MDSCs in the PBMCs, and the level of serum IL-10 and IL-12, IgE, EOS in normal healthy controls, pneumonia patients, attack asthma patients, and alleviated patients 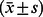.

Group	n	MDSCs (%)	IL-10 pg/ml	IL-12 pg/ml	IgE ug·L^−1^	EOS (×10^9^·L^−1^ )
normal healthy control	48	0.64±0.28	13.91±2.57	61.23±11.51	21.199±8.354	0.167±0.083
pneumonia	45	0.58±0.25	14.01±3.04	56.79±10.36	22.301±7.154	0.169±0.092
attach ashma	52	3.61±0.92 # * $	45.13±7.1# * $	35.33±8.5# * $	38.933±6.945# * $	0.291±0.201# * $
alleviated	50	1.15±0.38	12.83±3.26	58.45±9.23	12.83±3.26	0.176±0.098
*F*		1043.82	1284.47	1139.38	83.789	18.952
*P*		<0.05	<0.05	<0.05	<0.05	<0.05

# compared to the normal group;

* compared to the pneumonia group;

$ cmpared to the budesonide treated group.

### Pathological Changes in Asthmatic Mice and Budesonide Treated Asthmatic Mice Compared to Normal Controls

**Figure 3:**
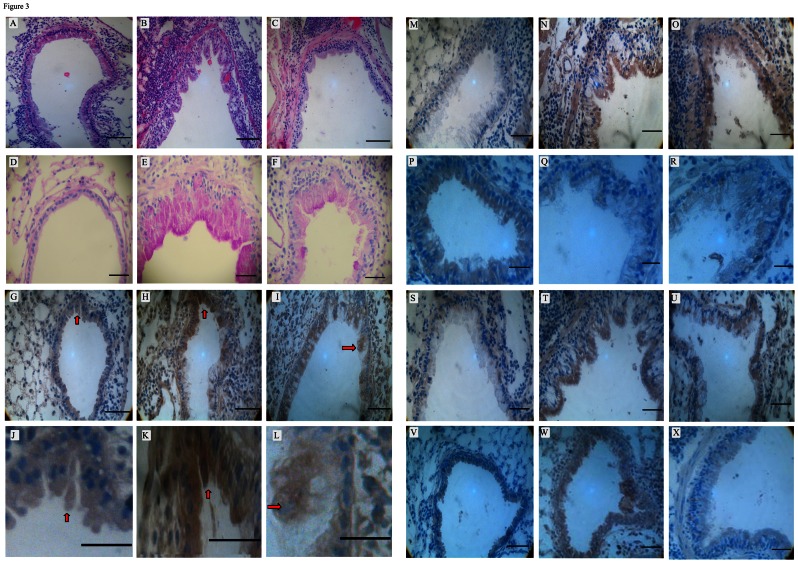
Different staining of the airway from normal controls (A, D, G, J, M, P, S, V), asthma mice (B, E, H, K, N, Q, T, W) and alleviated mice (C, F, I, L, O, R, U, X) and showed significant differences (Scale bars, 200 µm). (A–C), Airway H&E staining on mice from three groups shows significant difference (×200); (D–F), The PAS staining of distinct mice show significant difference; (G–I), MDSCs IHC staining on mice from three groups shows significant difference (×200), arrows indicate positive staining; (J–L), higher magnification of MDSCs IHC staining; (M–O), IL-10 IHC staining on mice from three groups shows significant difference; (P–R), IL-12 IHC staining on mice from three groups shows significant difference (×200); (S–U), TNF-α IHC staining on mice from three groups shows significant difference (×200); (V–X), IL-6 IHC staining on mice from three groups shows significant difference (×200).

To validate our conclusion from the patient study and to investigate the mechanism of asthma onset and the possible role of MDSCs, IL-10, and IL-12 during onset, we used mouse models. We established the asthmatic mouse model by treating the mice with OVA/aluminum and the alleviated asthmatic mouse model by co-treating the mice with budenoside. We first analyzed the pathological difference between normal control mice, asthmatic mice, and alleviated mice. H&E staining of lung tissue from the asthma mice showed severe infiltration of inflammatory cells (including EOS, neutrophils, and lymphocytes) into the bronchial submucosa, bronchial, and perivascular spaces. We also observed submucosal edema, mucous gland hyperplasia, and increased mucous secretion accompanied by increased mucosal folds, visible epithelial fracture, epithelial cell shedding, bronchiole smooth muscle mild hypertrophy, as well as bronchial wall and basement membrane thickening and shape irregularity, Similar, but milder, pathological changes were also observed in the lung tissue samples of the budesonide intervention group, but none of them were observed in the normal control group (Figure 3A–3C and Table 2). These results showed, the asthma mouse model was well-established and the alleviation treatment was also functional. Moreover, PAS staining on mouse tissues demonstrated more PAS-positive cells in asthma attack group compared to alleviated group and control group, which is consistent with H&E staining results (Table S1 and Figure 3D–3F).

**Table 2:** The thickness of smooth muscle layer, airway wall and epithelium mucous among three groups of mice (*µm*) 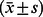.

Group	N	smooth muscle layer	airway wall	epithelium mucous
normal control	10	8.954±3.198	50.902±8.135	42.978±7.123
asthma mice	10	29.630±6.182*	153.148±25.879*	129.468±23.312*
alleviated	10	16.108±4.154* #	112.536±5.731* #	89.324±7.129* #
*F*		79.954	120.904	90.456
*P*		<0.05	<0.05	<0.05

* compared to normal control group, *P*<0.05;

# compared to asthma mice group, *P*<0.05.

### MDSCs and IL-10 were Upregulated, while IL-12 was Downregulated in Asthmatic Mice

The patient study indicated that accumulation of MDSCs and serum IL-10 are positively correlated and the accumulation of MDSCs and serum IL-12 are negatively correlated. This prompted us to ask how MDSCs, IL-10, and IL-12 levels are regulated in asthmatic mice and what the possible mechanism of asthma onset is. We first looked at the accumulation of MDSCs in different mice. MDSCs were mainly expressed in the cytosol of bronchus epithelial cells and lung parenchymatous cells, shown as brown granules inside the cells. The expression of MDSCs was significantly higher in the asthmatic mice 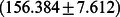 compared with the control mice (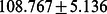, p<0.05) and was prominently down-regulated upon budesonide treatment (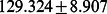, p<0.05) (Figure 3G–3L and Table 3).

**Table 3:** The level of MDSCs, IL-10, and IL-12, determined by IHC staining of lung tissues from the three groups of mice 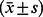.

Groups	n	MDSCs	IL-10	IL-12
normal control	10	108.767±5.136	100.939±6.879	151.467±6.991
asthma mice	10	156.384±7.612*	145.764±7.104*	109.754±7.910*
alleviated	10	129.324±8.907* #	129.378±5.331* #	137.903±8.512* #
*F*		348.902	87.12	110.998
*P*		<0.05	<0.05	<0.05

* compared with normal control group, *P*<0.05;

# Compared with asthma mice group, *P*<0.05.

We then analyzed the accumulation of IL-10 to study the possible mechanism of asthma onset in mice. IL-10 was mainly detected in bronchial epithelial cells, alveolar epithelial cells, macrophages, and neutrophils, shown as brown granules inside the cells. The asthmatic mice had higher expression of IL-10 (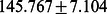) compared with the control mice (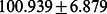, p<0.05), and the level of IL-10 was significantly down-regulated after budesonide treatment (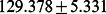, p<0.05) (Figure 3M–3O and Table 3).

The accumulation of IL-12 was also studied in mice. Mouse bronchial epithelial cells, alveolar epithelial cells, macrophages, and neutrophils were positive for granular IL-12 staining. The IL-12 expression in the asthmatic mice was much lower (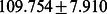) compared with the control mice (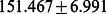, p<0.05) and was significantly increased after budesonide treatment (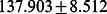, p<0.05,) (Figure 3P–3R and Table 3).

Thus, the results from the mouse models are consistent with those from the patient study. Taken together, these studies strongly suggest that MDSCs contribute to the onset and development of asthma by up-regulating the level of IL-10 and down-regulating the level of IL-12.

## Discussion

Cytokines and immune cells play important roles in the development of asthma, therefore it is significant for the diagnosis and treatment of asthma to identify effective biomarkers. Here, we found that the accumulation of MDSCs and IL-10 were induced in asthmatic children and mice, while the level of IL-12 was downregulated. Further statistical analysis showed that the accumulation of MDSCs and IL-10 are positively correlated, while the accumulation of MDSCs and IL-12 are negatively correlated. Thus, the accumulation of MDSCs may be the cause of IL-10 up-regulation and IL-12 down-regulation, thereby affecting asthma onset and development.

Since 1986, it has been well established that disruption of the balance between Th1 and Th2 activity can lead to hypersensitivity diseases such as bronchial asthma; however, the underlying mechanisms are still unclear [11]. Airway remodeling, often caused by unresolved, long-term airway inflammation and repeated stimulation of the wound healing process, is the pathological foundation of irreversible airflow limitation and sustained airway hyperresponsiveness during the pathogenesis of refractory asthma [12]. So far, responses to asthma treatments are still heterogeneous among refractory patients. Therefore, discovering novel markers in asthma pathogenesis and treatment targets has been a focus of asthma research.

MDSCs, including immature macrophages, granulocytes, dendritic cells, and other progenitor stage myeloid cells have recently been discovered as novel tumor markers. This study demonstrated significant increased BALF in the asthma group compared to alleviated group and control group, including neutrophils, eosinophils, macrophages, and lymphocytes (Table S2). This accumulation of inflammatory cells suggests that asthma is a type of chronic airway inflammation.

MDSCs overexpress ArgI and Inducible Nitric Oxide Synthase (iNOS), deplete L-arginine, and eventually suppress the activation of T cells [13]. Meanwhile, the NO produced from L-arginine depletion not only suppresses the expression of Major Histocompatibility Complex (MHC) II molecules and MHC II-mediated T cell apoptosis but also dilates blood vessels and promotes inflammatory cell infiltration and inflammatory mediator release. The low L-arginine environment in MDSCs promotes the transformation of iNOS into reactive oxygen species (ROS), which recruits and activates immune effector cells, promotes cytokines release, and causes airway smooth muscle spasm, thereby leading to the onset of asthma [14]. MDSCs have been demonstrated to mediate lung inflammation and airway hyperresponsiveness through production of ROS, suggesting multiple mechanisms through which MDSCs cause asthma [15]. Our study discovered elevated level of serum NO, Arg I and cellular ROS during the onset of asthma, which is consistent with their synergistic effect in asthma development. Our study showed that the percentage of MDSCs in asthma patients was significantly higher compared to the normal controls, budesonide-treated asthma patients, and pneumonia patients, while the latter three groups showed no significant differences. The percentage of MDSCs in asthmatic mice was significantly higher compared to the normal controls and budesonide-treated group, whereas there was no significant difference between the normal controls and asthma mice treated with budesonide. This indicates that MDSCs play a role in the onset of asthma, and their numbers are reduced in alleviated asthma. The difference in MDSCs levels in the asthma patients and the pneumonia patients suggest there is a difference between asthma which is a specific type of airway inflammation, and common airway bacterial infections. The published data indicated that monocytic (CD11b+Ly-6G-Ly-6GC high) MDSC increases IL-10 production through monocyte-dependent STAT1, iNOS and NO production, whereas granulocytic (CD11b+Ly6G+Ly-6C low) MDSC does this by a granulocyte-dependent ARG and ROS pathway [13], [16], [17], [18], [19], [20]. We analyzed the accumulation of NO and ARG/ROS, Table S3, and found that both the accumulation of NO and ARG/ROS increased in the asthma mice and the accumulation of them are all prominently down-regulated upon budesonide treatment. Therefore, we suspect that both granulocytic (CD11b+Ly6G+Ly-6C low) and monocytic (CD11b+Ly-6G-Ly-6GC high) MDSCs contribute to the production of IL-10 during the onset of asthma.

IL-10 is a widely distributed multifunctional cytokine that plays a dual role in inflammation. Studies have shown that IL-10 not only suppresses the production of proinflammatory factors by macrophages but also reduces cytotoxic effect and decreases NO production. Reduced IL-10 production increases the production of proinflammatory cytokines, leading to chronic inflammation, airway remodeling, airflow limitation, and lung tissue damage [16]. Gene polymorphism in IL-10 has been reported to play an important role in the onset of asthma [17]. Asthma patients treated with antigen specific immunotherapy showed increased IL-10 and suppressed T cells; however, T cell proliferation and cytokine secretion was completely restored upon neutralization of IL-10 in the peripheral blood of these asthma patients [18]. These results suggest the important role of IL-10 in the inflammatory response during the onset of asthma. IL-12 is an essential cytokine for Th1 cell generation and functions by promoting the differentiation of Th1 cells from Th0 cells while suppressing Th2 cell differentiation. IL-12 suppresses the onset and progression of allergies. IL-12 plays an important role in the onset of asthma, and the gene polymorphism of IL-12 is correlated to the risk index of asthma [19]. It has been showed that the IL-10 level in both asthmatic group and alleviated group was lower than control group [20], while another study reported that the IL-10 level in asthmatic children without a family history of asthma was comparable with that in healthy control group [21]. However, our study showed that asthmatic children expressed higher levels of IL-10 and lower levels of IL-12 compared to normal controls, budesonide-treated asthma patients, and pneumonia patients, while the latter three demonstrated no significant differences (Table 1). The study on asthma induced by virus infection demonstrated high level of IL-10 during disease onset, which is consistent with our observations [22].

It is speculated that the level of IL-10 in asthma onset is correlated with the cause of diseases. High level of IL-10 is often observed in virus-induced asthma attack, which is consistent with the elevated IL-10 we observed in this study, as many cases in the asthma attack group was detected positive for respiratory virus (Respiratory Syncytial Virus and Rhinovirus). The young age of research objects in this study may have also accounted for the high expression of IL-10. Higher IL-10 and lower IL-12 levels were also observed in the asthmatic mice compared to normal controls and budesonide-treated mice, while the latter two demonstrated no significant difference. These results indicate the changes in IL-10 and IL-12 levels during the onset and alleviation of asthma. The increased IL-10 and decreased IL-12 in asthma children indicates the loss of balance in Th1/Th2 cells, confirming the important role of Th1/Th2 balance in the mechanism of asthma pathogenesis. Our study also suggests that asthma is specific type of airway inflammation that is different from common airway bacterial infections.

Severe inflammation induces the production of IL-10 by MDSCs, which down-regulates the level of IL-12 [8], therefore we speculate that MDSCs plays an important role in the onset of asthma through up-regulating IL-10 and down-regulating IL-12. What is the role of MDSCs? It has been reported that IL-10 has important functions in the regulation of MDSCs, as IL-10 induces the production of ROS, which is generated by Nicotinamide adenine dinucleotide phosphate (NADPH) oxidase and mediates the suppression of T cells by MDSCs [20], [21], [22]. The induction of regulatory T cells (Tregs) depends on the production of IL-10 and transforming growth factor (TGF)-β, or IL-10 and arginase from a subpopulation of MDSCs [23]. We found that Treg cells/CD4^+^ Tcells were mainly expressed in alveolar epithelial cells and macrophages, identified as brown granular staining. The Treg cells/CD4^+^ Tcells were significantly increased in the asthma attack group (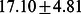
%) compared to the control groups (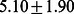
5%), and were prominently reduced upon Budesonide treatment (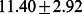, p<0.05%) (Table S4). This indicates the important regulatory role of Tregs in the onset of asthma. Currently, the role of IL-10 in asthma pathogenesis is still under debate. A high level of IL-10 was observed in virus-induced asthma; however, IL-10 was reported to inhibit the onset of asthma by inhibiting Th2 cytokine production as well as eosinophil infiltration, and the reduction of IL-10 and the increase of IL-4/IFN-γ (interferon-gamma) lead to the onset of severe asthma [23], [24], [25]. Our study also discovered that the serum levels of IL-4, IL-17 and IL-13 (Table S5) in the asthma group were significantly higher than those in the alleviated group and control groups. In addition, the expression of TNF-α and IL-6 (Table S6, Figure 3S–3U and 3V–3X) in the lung tissue of asthmatic mice were also significantly increased compared to the alleviated group and control groups. These data indicate the involvement of multiple cytokines in the development of asthma. Most of the asthmatic children in our study had asthma which was induced by an airway viral infection, which could be associated with the high expression of IL-10. MDSCs has been reported to play a suppressive role through down-regulating the level of IL-12 secreted by macrophages, and other studies demonstrated that a high level of IL-10 produced by MDSCs under severe inflammation reduces the secretion of IL-12 by macrophages [8], [24]. Thus, inflammation induces the MDSCs to secrete IL-10, which down-regulates the secretion of IL-12 by macrophages through a cell surface Toll-like receptor (TLR4) dependent mechanism. Our study showed that IL-10 and MDSCs were highly expressed in asthmatic children, whereas IL-12 expression was low compared to normal controls. Further investigation indicate that the MDSC percentage in PBMCs is positively correlated with the level of IL-10 and negatively correlated with the level of IL-12, which is consistent with the role of IL-10 in reducing the level of IL-12.

Cytokines, as well as their positive and negative regulatory network, play important roles in the pathogenesis of asthma. Airway remodeling during asthma pathogenesis, including airway wall thickening, matrix deposition, collagen deposition, subepithelial fibrosis, airway smooth muscle hyperplasia and hypertrophy, myofibroblast hyperproliferation, mucous gland and goblet cell hyperplasia, thickening of reticular layer under epithelium, and capillary angiogenesis, is the consequence of repeated stimulation by cytokines and growth factors [25]. Studies demonstrate that airway remodeling, a major pathological characteristic of bronchial asthma, is closely related to the incomplete reversible airflow limitation and airway hyperresponsiveness. In a liver cancer mouse model, MDSCs in the spleen, peripheral blood, lymph nodes, and tumor region secrete a large amount of IL-10, which suppresses the production of IL-12 by MDSCs and thereby suppresses the activation of T cells by MDSCs [26]. The inflammation-induced interaction between MDSCs and macrophages is dependent on TLR4, as TLR4^−/−^ mice MDSCs are impaired in IL-10 production and cannot reduce the production of IL-12 by macrophages, resulting in impaired Supression of activation of T cells in spite of the severity of inflammations [8], [27], [28]. Our mouse models showed that the asthma group had significantly thicker airway walls and airway smooth muscle layers, damaged epithelium, abrupt ending of mucous, narrow bronchus lumen, and leukocyte infiltration under the airway wall mucous and around the airway wall. The asthma group mice exhibited significantly higher levels of MDSCs and IL-10 than in the normal control mice, and budesonide treatment greatly reduced their expressions. In contrast, IL-12 expression in the asthmatic mice was significantly lower than in normal controls and was increased after budesonide treatment. These results are consistent with previous findings, demonstrating the correlation between MDSCs, IL-10, and IL-12 levels and the onset and severity of asthma disease.

Our data from asthma patients and asthma mouse models indicate that the interaction between MDSCs and macrophages leads to a significant increase in the levels of IL-10 and decrease in levels of IL-12, which promote the development of chronic inflammation seen in asthma. Thus, MDSCs may potentially be a novel target in the treatment of asthma. Further study will be necessary for elucidating the precise role of MDSCs in the pathogenesis of asthma and developing efficient therapeutics.

## Supporting Information

Table S1
**Quantitation of PAS-positive cells.** Quantitation of PAS-positive cells in the lung tissue of mice from three groups 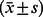.(DOC)

Table S2
**Quantitation of BALF and other cells.** Quantitation of BALF cells, neutrophils, and eosinophils in mice from three groups. 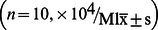

(DOC)

Table S3
**Accumulation of NO Arginase and ROS.** Serum content of NO 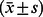 (umol·L^−1^), Arginase I (OD) and intracellular ROS (%) in mice from three groups.(DOC)

Table S4 **Accumulation of RALF MAC cell.** Lung BALF MAC cell counting, and ratio of Gr-1^+^CD11b^+^MDSCs as well as CD25^+^Tregs over CD4^+^T cells in mice from three groups.
(DOC)

Table S5
**Accumulation of IL-13, IL-4 and IL-17.** Serum IL-13, IL-4 and IL-17 levels in mice from three groups 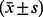 (ng·L^−1^).(DOC)

Table S6 **Expression levels of IL-6 and TNF-α.** Expression levels of IL-6 and TNF-**α** in the lung tissue of mice from three groups.
(DOCX)
