# Supplementary material for: Peripheral Blood MDSCs, IL-10 and IL-12 in Children with Asthma and Their Importance in Asthma Development
Source: PLoS One. 2013 May 22;8(5):e63775. doi: 10.1371/journal.pone.0063775 (PMC3661689; doi:10.1371/journal.pone.0063775)
Supplement: Table S1 — Quantitation of PAS-positive cells. Quantitation of PAS-positive cells in the lung tissue of mice from three groups . (DOC) [file pone.0063775.s001.doc]

**Table S1.**

**Quantitation of PAS-positive cells: Quantitation of PAS-positive cells in the lung tissue of mice from three groups (±*s*).**

| groups | n | PAS positive cell |
| --- | --- | --- |
| normal control | 10 | 67.356±10.248 |
| asthma mice | 10 | 134.147±31.908^*^ |
| alleviated | 10 | 93.145±13.754^*#^ |
| *F* |  | 109.731 |
| *P* |  | <0.05 |

^*^: compared with normal control group, *P*<0.05; ^#^: Compared with asthma mice group, *P*<0.05
