# Supplementary material for: Peripheral Blood MDSCs, IL-10 and IL-12 in Children with Asthma and Their Importance in Asthma Development
Source: PLoS One. 2013 May 22;8(5):e63775. doi: 10.1371/journal.pone.0063775 (PMC3661689; doi:10.1371/journal.pone.0063775)
Supplement: Table S2 — Quantitation of BALF and other cells. Quantitation of BALF cells, neutrophils, and eosinophils in mice from three groups. (DOC) [file pone.0063775.s002.doc]

**Table S2.**

**Quantitation of BALF and other cells:** Quantitation of BALF cells, neutrophils, and eosinophils in mice from three groups (n=10,×10^4^/Ml $\bar{x}$±s).

| Groups | n | neutrophils | eosinophils | macrophages | lymphocytes |
| --- | --- | --- | --- | --- | --- |
| normal control | 10 | 2.36±0.91 | 1.71±0.87 | 0.83±0.20 | 1.21±0.45 |
| asthma mice | 10 | 13.28±3.17^*^ | 10.19±2.57^*^ | 5.89±1.09^*^ | 6.57±1.24^*^ |
| alleviated | 10 | 7.81±2.24^*#^ | 5.23±1.49^*#^ | 3.24±0.87^*#^ | 4.23±1.29^*#^ |
| *F* |  | 267.451 | 253.424 | 378.923 | 223.031 |
| *P* |  | *P*<0.05 | *P*<0.05 | *P*<0.05 | *P*<0.05 |

^*^: compared with normal control group, *P*<0.05; ^#^: Compared with asthma mice group, *P*<0.05
