# Supplementary material for: Peripheral Blood MDSCs, IL-10 and IL-12 in Children with Asthma and Their Importance in Asthma Development
Source: PLoS One. 2013 May 22;8(5):e63775. doi: 10.1371/journal.pone.0063775 (PMC3661689; doi:10.1371/journal.pone.0063775)
Supplement: Table S3 — Accumulation of NO Arginase and ROS. Serum content of NO (umol·L−1), Arginase I (OD) and intracellular ROS (%) in mice from three groups. (DOC) [file pone.0063775.s003.doc]

**Table S3.**

**Accumulation of NO Arginase and ROS:** Serum content of NO ($\bar{x}$±s) (umol·L^-1^), Arginase I (OD) and intracellular ROS (%) in mice from three groups.

| Groups | n | serum NO (umol·L^-1^) | Arg I (OD) | Intracellular ROS(%) |
| --- | --- | --- | --- | --- |
| normal control | 10 | 31.23±9.74 | 0.123±0.007 | 81.24±11.54 |
| asthma mice | 10 | 97.94±20.35^*^ | 0.358±0.032^*^ | 227.43±35.42^*^ |
| alleviated | 10 | 50.45±12.43^*#^ | 0.204±0.011^*#^ | 136.94±17.28^*#^ |
| *F* |  | 101.457 | 71.232 | 129.38 |
| *P* |  | <0.05 | <0.05 | <0.05 |

*: compared with control group, *P*<0.05; ^#^: compared with asthma group, *P*<0.05
