# Supplementary material for: Peripheral Blood MDSCs, IL-10 and IL-12 in Children with Asthma and Their Importance in Asthma Development
Source: PLoS One. 2013 May 22;8(5):e63775. doi: 10.1371/journal.pone.0063775 (PMC3661689; doi:10.1371/journal.pone.0063775)
Supplement: Table S4 — Accumulation of RALF MAC cell. Lung BALF MAC cell counting, and ratio of Gr-1+CD11b+MDSCs as well as CD25+Tregs over CD4+T cells in mice from three groups. (DOC) [file pone.0063775.s004.doc]

**Table S4.**

**Accumulation of RALF MAC cell:** Lung BALF MAC cell counting, and ratio of Gr-1^+^CD11b^+^MDSCs as well as CD25^+^Tregs over CD4^+^T cells in mice from three groups.

| Groups | n | MDSCs (%) | MAC（×10^4^/Ml） | Tregs (%) |
| --- | --- | --- | --- | --- |
| normal control | 10 | 1.12±0.61 | 0.83±0.20 | 5.10±1.90 |
| asthma mice | 10 | 5.32±2.18^*^ | 5.89±1.09^*^ | 17.10±4.81^*^ |
| alleviated | 10 | 3.41±1.42^*#^ | 3.24±0.87^*#^ | 11.40±2.92^*#^ |
| *F* |  | 34.78 | 123.21 | 23.19 |
| *P* |  | <0.05 | <0.05 | <0.05 |

^*^: compared with normal control group, *P*<0.05; ^#^: compared with asthma mice group, *P*<0.05
