# Supplementary material for: Peripheral Blood MDSCs, IL-10 and IL-12 in Children with Asthma and Their Importance in Asthma Development
Source: PLoS One. 2013 May 22;8(5):e63775. doi: 10.1371/journal.pone.0063775 (PMC3661689; doi:10.1371/journal.pone.0063775)
Supplement: Table S5 — Accumulation of IL-13, IL-4 and IL-17. Serum IL-13, IL-4 and IL-17 levels in mice from three groups (ng·L−1). (DOC) [file pone.0063775.s005.doc]

**Table S5.**

**Accumulation of IL-13, IL-4 and IL-17:** Serum IL-13, IL-4 and IL-17 levels in mice from three groups (x±s) (ng·L^-1^).

| Group | n | IL-13（ng·L^-1^） | IL-4（ng·L^-1^） | IL-17（ng·L^-^） |
| --- | --- | --- | --- | --- |
| normal healthy control | 48 | 77.27±18.16 | 18.97±4.98 | 3.97±1.78 |
| pneumonia | 45 | 80.71±18.08 | 8.14±2.74 | 4.58±2.77 |
| attach ashma | 52 | 164.44±21.02^#^*^$^ | 42.38±15.67^#^*^$^ | 8.76±2.13^#^*^$^ |
| alleviated | 50 | 86.08±20.88 | 23.91±5.14 | 4.15±1.82 |
| *F* |  | 210.371 | 198.352 | 174.234 |
| *P* |  | <0.05 | <0.05 | <0.05 |

^#^: compared with the normal group; *: compared with the pneumonia group; ^$^: compared with the budesonide treated group
