# Supplementary material for: Peripheral Blood MDSCs, IL-10 and IL-12 in Children with Asthma and Their Importance in Asthma Development
Source: PLoS One. 2013 May 22;8(5):e63775. doi: 10.1371/journal.pone.0063775 (PMC3661689; doi:10.1371/journal.pone.0063775)
Supplement: Table S6 — Expression levels of IL-6 and TNF-α. Expression levels of IL-6 and TNF-α in the lung tissue of mice from three groups. (DOCX) [file pone.0063775.s006.docx]

**Table S6.**

**Expression levels of IL-6 and TNF-α:** Expression levels of IL-6 and TNF-**α** in the lung tissue of mice from three groups.

| Groups | n | IL-6 | TNF-α |
| --- | --- | --- | --- |
| normal control | 10 | 103.847±6.453 | 123.756±10.124 |
| asthma mice | 10 | 153.456±8.673^*^ | 150.782±20.109^*^ |
| alleviated | 10 | 128.354±6.124^*#^ | 135.414±9.476^*#^ |
| *F* |  | 102.678 | 90.442 |
| *P* |  | <0.05 | <0.05 |

^*^: compared with normal control group, *P*<0.05; ^#^: compared with asthma mice group, *P*<0.05
